# Tuberculosis (TB) incidence in a cohort of individuals infected with human T-lymphotropic virus type 1 (HTLV-1) in Salvador, Brazil

**DOI:** 10.1186/1742-4690-12-S1-P37

**Published:** 2015-08-28

**Authors:** Maria Fernanda Rios Grassi, Normeide Pedreira dos Santos, Monique Lírio, Afrânio Lineu Kritski, Maria Conceição Almeida Chagas, Leonardo Pereira Santana, Noilson Lázaro, Juarez Dias, Bernardo Galvão-Castro

**Affiliations:** 1 Centro Integrativo e Interdisciplinar de HTLV (CHTLV), Escola Bahiana de Medicina e Saúde Pública,; Salvador, Bahia, Brasil; 2 Laboratório Avançado de Saúde Pública, Centro de Pesquisas Gonçalo Moniz, Fundação Oswaldo Cruz, Salvador, Bahia, Brasil; 3 Hospital Universitário Professor Edgar Santos, Salvador Bahia, Brazil; 4 Programa Acadêmico de Tuberculose, Faculdade de Medicina; Universidade Federal do Rio de Janeiro, Rio de Janeiro, Brazil; 5 Departamento de Vigilância Epidemiológica - Secretaria da Saúde do Estado da Bahia, Salvador, Bahia, Brazil

## 

Few reports evaluate the association between HTLV-1 and TB in countries where both infections are endemic. The incidence and relative risk of TB in a cohort of HTLV-1-infected individuals was estimated from 22 to 2012. Using records from the CHTLV database, the Information System on Diseases of Compulsory Declaration (Sinan) database was searched for TB cases matching each patients’ full name, date of birth and mother's full name. Structured Query Language for use in relational databases was used to cross match both registries by employing a “linkage” strategy. TB incidence density (ID) in both seropositive and seronegative HTLV-1 subjects was calculated as the number of new-TB-cases per 1,0 person-years of follow-up. From a cohort of 6,495 patients, 1,711 were infected with HTLV-1. 73 TB cases occurred during the 11-year study period: 33 in HTLV-1-infected patients and 40 in uninfected individuals. The ID for the HTLV-1/TB group was 3.3 per 1,0 person-years and 1.1 per 1,0 person-years in the non-infected individuals. The relative risk for TB in HTLV-1+ group was 2.3 (CI 95% 1.46 - 3.65). In addition, HTLV-1-infected individuals aged 31 to 50 presented the highest ID (3.4). In this group, the relative risk for TB was 2.6 (CI 95% 1.2-5.5). HTLV-1 patients with TB were older (p = 0. 5), had lower educational levels (p=0.02) and presented higher recurrence rates of TB (p=0.09) than non-HTLV-infected individuals with TB. HIV serology was positive for 6.0% of individuals infected with HTLV-1 versus 12.5% of individuals with isolated TB (p= 0.45). Patients infected with HTLV-1 are more susceptible to TB. The populations of HTLV/TB co-infected patients and HTLV-1-infected individuals share similar epidemiological features, highlighting the neglected nature of both diseases.

